# High Preoperative Body Mass Index Is Associated With Implant Breakage in Patients Treated With Magnetically Controlled Growing Rods for Early-onset Scoliosis

**DOI:** 10.1097/BPO.0000000000002988

**Published:** 2025-05-05

**Authors:** Antti J. Saarinen, Lindsay Andras, Oheneba Boachie-Adjei, Patrick Cahill, Tenner Guillaume, Brian Snyder, Paul Sponseller, Peter Sturm, Michael Vitale, Ilkka Helenius, Pediatric Spine Study Group

**Affiliations:** Departments of * Paediatric Orthopaedic Surgery; † Orthopaedics and Traumatology, University of Turku and Turku University Hospital, Turku; ‡ Spine Surgery, Childrens’ Hospital Los Angeles, Los Angeles, CA; § FOCOS Orthopedic Hospital Accra, Accra, Ghana; ∥ Department of Orthopaedic Surgery, Children’s Hospital of Philadelphia, Philadelphia, PA; ¶ Department of Orthopaedics, Gillette Children’s, St Paul, MN; # Department of Orthopedic Surgery, Boston Children’s Hospital, Boston, MA; ** Division of Pediatric Orthopaedics, Johns Hopkins University, Baltimore, MD; †† Cincinnati Children’s Hospital Medical Center, Cincinnati, OH; ‡‡ Department of Orthopedics and Traumatology, Helsinki University Hospital, Helsinki, Finland

**Keywords:** body mass index, obesity, scoliosis

## Abstract

**Introduction:**

Magnetically controlled growing rods (MCGRs) have become the current standard in the growth-friendly treatment of patients with early-onset scoliosis (EOS). MCGRs allow noninvasive lengthenings with external lengthening device and reduce the need for surgical procedures. The association of preoperative body mass index (BMI) and the outcomes of the MCGR treatment is not well known.

**Methods:**

Prospectively collected international database was reviewed for EOS patients treated with MCGR. Patients without preoperative BMI data or follow-up <2 years were excluded. Patients were classified as healthy weight, overweight, and underweight using Centers for Disease Control and Prevention (CDC) growth charts. Quality of life was assessed using EOSQ-24. Results were analyzed from the 2-year follow-up.

**Results:**

A total of 663 patients were categorized into underweight (n=91), healthy weight (n=417), and overweight (n=155) groups. There were no significant differences in major curve correction or thoracic height increase among the BMI groups, irrespective of etiology. Distribution of BMI categories differed significantly by etiology (*P*=0.009), with lower healthy weight proportions in the syndromic group (92/167, 55%) compared with idiopathic (131/177, 74%) (adjusted *P*=0.004), and a higher underweight proportion in neuromuscular (36/244, 15%) compared with idiopathic (15/177, 8.5%) (adjusted *P*=0.044). Higher BMI z-scores were associated with an increased incidence of complications, including implant-related complications (RR 1.1, 95% CI 1.0-1.3) and implant breakage (RR 1.3, 95% CI 1.1-1.7). Healthy weight and underweight patients experienced lower overall complication rates compared with overweight patients. Implant-related complications were less common in underweight patients compared with overweight patients (RR 0.45, 95% CI 0.20-0.90). Higher BMI z-score was a significant predictor of implant breakage, whereas preoperative major curve, kyphosis, and etiology were not. EOSQ-24 scores did not differ significantly among BMI groups, and changes in scores were comparable across groups during follow-up.

**Conclusion:**

BMI status did not influence curve correction, thoracic height increase, or EOSQ-24 outcomes in early-onset scoliosis patients. However, the higher incidence of implant breakage in overweight patients suggests that elevated BMI should be carefully considered when planning treatment.

**Level of Evidence:**

Level III.

Early-onset scoliosis (EOS) refers to scoliosis diagnosed 9 years of age or younger and is further divided into idiopathic, neuromuscular, syndromic, and congenital scoliosis.^1^ EOS is frequently associated with comorbidities, which may affect the development and growth of the child. Certain syndromes like Prader-Willi syndrome are linked to obesity, whereas complex neuromuscular disorders can result in low BMI.^2^ Malnutrition in patients with EOS can be multifactorial, arising from poor oral intake, increased metabolic demands, and the mechanical effects of severe spinal deformity on gastrointestinal function. Complicated spinal deformity may lead to thoracic insufficiency syndrome (TIS), which causes increased work of breathing and increased energy consumption, leading to decreased weight.^3^ Growth-friendly treatment of EOS can increase patient weight in underweight patients.^4–7^

Modern surgical treatment aims to prevent the progression of the deformity while allowing the growth of the thoracic spine. Magnetically controlled growing rods (MCGRs) have become the current standard of surgical treatment in recent years. MCGRs allow noninvasive lengthening of the instrumentation using an external lengthening device.^8^ It is hypothesized that increased soft tissue depth may complicate the external lengthening of the MCGR instrumentation in patients with obesity.^9–11^ Manufacturer guidelines recommend that MCGRs not be implemented in patients with a BMI over 25 due to concerns about effective distraction through thicker soft tissue. MCGR implantation can also be challenging in extremely thin patients due to the relatively large size of the actuator component, which may lead to skin irritation or prominence. The use of MCGRs in overweight patients remains controversial, with no clear consensus on when obesity should be considered a contraindication.^12^

This study aims to assess the association between weight status and health-related quality of life in patients with EOS. In addition, we examine the rate of complications, the degree of deformity correction, thoracic height gain, and their relationship with BMI status. We hypothesize that underweight patients may be more susceptible to soft tissue-related complications and unplanned revisions. In addition, we propose that MCGR lengthening may be less effective, and the risk of implant-related complications may be higher in overweight patients.

## METHODS

Institutional review board approval was received from the University of California, San Diego, USA (no. 150962 R). A retrospective review of a prospectively collected international database was performed for patients treated with bilateral MCGRs (Magec, NuVasive Specialized Orthopedics, Inc., San Diego, CA) for EOS, with a minimum follow-up of 2 years after the initial surgery. Patients with conversion to MCGRs were excluded. Radiographic data was assessed preoperatively, after the initial surgery, and at 2-year follow-up. Major curves, kyphosis, thoracic height, weight and standing height, arm span, age, sex, and etiology were collected and analyzed. Complications and revision surgeries were assessed from the 2-year follow-up. Complications included implant-related, neurologic, and wound-related issues. A local fusion over the proximal and distal foundations was performed. Pseudoarthrosis refers to the lack of union around these foundations.

BMI is a limited measure for pediatric weight status as it does not account for age-specific and sex-specific growth patterns, body composition differences, and developmental variations. Therefore, patient weight status was assessed using the Centers for Disease Control and Prevention (CDC) growth charts, incorporating age, weight, height, and sex. Patients with a BMI Z-score below −2 were classified as underweight, between −2 and 1 as healthy weight, and above 1 as overweight.^13^ A strong positive correlation was observed between BMI z-scores and BMI status groups (r=0.86, 95% CI: 0.84-0.88, *P*<0.001). Statistical analysis was conducted in R 4.4.0 using the cdcanthro package (https://github.com/CDC-DNPAO/CDCAnthro) Linear mixed-effects models were used to assess the association between BMI status and the difference between the planned and actual distraction values over time, with BMI status and time since surgery as fixed effects and patient as a random effect to account for repeated measures. The incidence of complications and revisions were analyzed using single and multivariate Poisson regression models and reported using rate ratios (RRs) with 95% CIs. Planned surgical interventions included the exchange of magnetically controlled growing rods (MCGRs) upon reaching their maximum distraction capacity or performing a final fusion after completing the distraction period. The planned revision procedures did not include routine removal of the MCGRs according to the manufacturer's guidelines. Unplanned surgical interventions encompassed revisions for broken implants, fixation point failures, replacement of MCGRs that failed to lengthen, and irrigation and debridement for deep surgical site infections.

Patient-reported outcome was assessed using the Early Onset Scoliosis Questionnaire 24 (EOSQ-24) instrument preoperatively, at 6 months, and at 2 years after the initial surgery. EOSQ-24 is a disease-specific outcome measure.^14^ EOSQ-24 consists of Health-Related Quality of Life, Parental Burden, Financial Burden, and Satisfaction domains. Statistical analyses were conducted across BMI status groups for each domain at all time points, and for the change from preoperative to 2 years postoperatively.

## RESULTS

In all, 91 patients with underweight status (mean BMI 12.3, SD 1.0), 417 patients with a healthy weight status (mean BMI 15.5, SD 1.3), and 155 with overweight status (mean BMI 20.3, SD 3.0) were identified from the database (Table 1). There was no difference in major curve correction between the healthy status group (mean 31%, SD 20), underweight status group (mean 35%, SD 17), or overweight status group (mean 31%, SD 18). Thoracic height increase was median 5.0 mm (IQR 11 mm) in the healthy weight group, median 3.9 mm (IQR 14 mm) in the overweight group, and median 5.3 mm (IQR 8.3 mm) in the underweight group (*P*=0.450). When analyzed by etiology, there were no differences between the BMI status groups in the major curve correction or in the achieved thoracic height increase during the follow-up (Table 1). The linear mixed-effects models showed that time since surgery was significantly associated with an increase in the difference between planned and actual distraction values for both rods (*P*<0.001). However, BMI status was not significantly associated with the distraction differences.

**TABLE 1:** Patient Characteristics

	Healthy weight	Overweight	Underweight
n	417	155	91
Age	7.0 (1.8)	6.5 (2.1)	7.0 (1.8)
Female, n (%)	245 (58.8)	89 (57.4)	55 (60.4)
Preoperative major curve angle	74 (20)	77 (20)	72 (22)
Preoperative weight (kg)	20.0 (5.4)	24.5 (10.1)	16.5 (3.8)
Preoperative height (cm)	112.8 (13.5)	108.1 (17.8)	114.8 (13.1)
Preoperative arm span (cm)	114.2 (16.3)	117.9 (19.4)	110.1 (14.1)
Preoperative body mass index (BMI)	15.5 (1.3)	20.4 (3.0)	12.3 (1.0)
Mean BMI Z-score	−0.2 (0.8)	1.7 (0.7)	−3.7 (1.6)
Idiopathic etiology, n (%)	131 (31.4)	31 (20.0)	15 (16.5)
Congenital etiology, n (%)	49 (11.8)	18 (11.6)	8 (8.8)
Syndromic etiology, n (%)	92 (22.1)	43 (27.7)	32 (35.2)
Neuromuscular etiology, n (%)	145 (34.8)	63 (40.6)	36 (39.6)
Postoperative major curve angle	41 (17)	42 (16)	38 (15)
Major Curve angle 2 y postoperatively	42 (18)	46 (16)	38 (17)
Preoperative T1-T12 height (mm)	166 (30)	163 (31)	167 (27)
Postoperative T1-T12 height (mm)	191 (30)	192 (32)	191 (20)
T1-T12 height at 2-y follow-up (mm)	201 (29)	197 (34)	200 (2)

All values given as means and SDs or sum and percentages.

In the idiopathic group, 131 patients (74%) were classified as healthy weight, 15 patients (9%) as underweight, and 31 patients (18%) as overweight. Of all patients with neuromuscular EOS, 145 patients (59%) had healthy weight status, 36 patients (15%) had underweight status, and 63 patients (26%) had overweight status. In the syndromic group, 92 patients (55%) had healthy weight status, 32 patients (19%) had underweight status, and 43 patients (26%) had overweight status. In the congenital group, 49 patients (65%) had healthy weight status, 8 patients (11%) had underweight status, and 18 patients (24%) had overweight status. The relationship between etiology and the distribution of the BMI status groups was statistically significant [χ² (6, N=663)=17.12, *P*=0.009]. Pairwise comparisons after Bonferroni correction revealed significant differences in BMI category distributions between the idiopathic and syndromic groups (adjusted *P*=0.004), caused by a lower proportion of healthy weight individuals in the syndromic group (92/167, 55%) compared with the idiopathic group (131/177, 74%). In addition, significant differences were observed between the idiopathic and neuromuscular groups (adjusted *P*=0.044), with the neuromuscular group showing a higher proportion of underweight individuals (36/244, 15%) compared with the idiopathic group (15/177, 8.5%).

**FIGURE 1:**
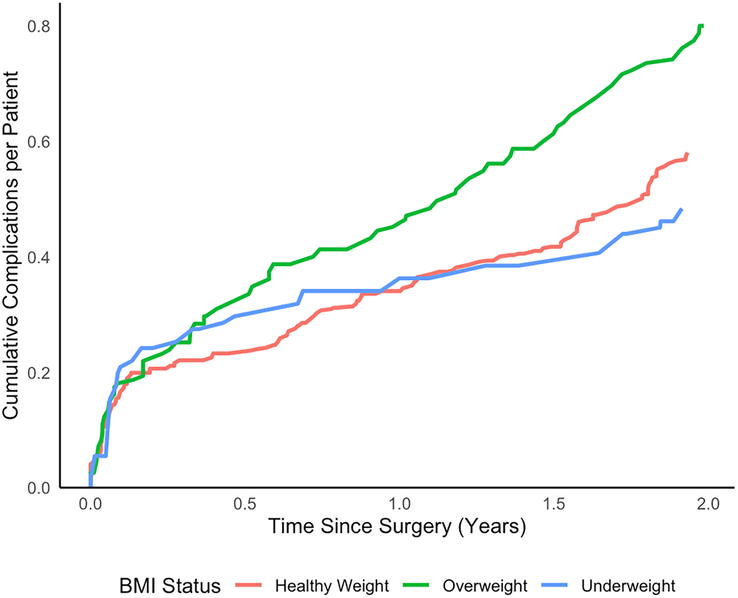
Cumulative sum of total complications per patient for each group during the 2-year follow-up.

Higher BMI z-score was associated with increased incidence of total number of complications (RR 1.1, 95% CI 1.1-1.2), implant-related complications (RR 1.1, 95% CI 1.0-1.3), and implant breakage (RR 1.3, 95% CI 1.1-1.7) (Table 2). The total incidence of complications was lower in healthy weight (RR 0.72, 95% CI 0.59-0.89) and underweight patients (RR 0.65, 95% CI 0.47-0.89) when compared with overweight patients (Fig. 1, Table 3). Implant-related complications were more common in overweight patients when compared with underweight patients (RR 0.45, 95% CI 0.20-0.90). Implant breakage was less common in healthy weight (RR 0.37, 95% CI 0.18-0.79) and underweight patients (RR 0.12, 95% CI 0.01-0.6) when compared with overweight patients. In the healthy weight group, the incidence of wound-related complications (RR 0.53, 95% CI 0.31-0.94), dehiscence (RR 0.33, 95% CI 0.14-0.80), and superficial wound infections (RR 0.33, 95% CI 0.11-0.91) were less common than in the overweight patients. Device migration, anchor prominence, MCGR actuator malfunction, MCGR failure to lengthen, and neurological complications did not differ between the BMI status groups. A multivariate analysis was performed to further analyze potential predictors of implant breakage using BMI z-score, preoperative major curve, preoperative maximum sagittal kyphosis, and etiology. The results showed that BMI z-score was a significant predictor (*P*=0.010), whereas other factors were not statistically significant. There was no observed association between higher BMI z-score within the overweight group and the incidence of complications.

**TABLE 2:** The Association of BMI Z-score and Complications During the 2-year Follow-up

Complication	Rate ratio	Lower CI	Upper CI	Significance
Total complications	1.11	1.05	1.18	0.00
Implant related	1.13	1.01	1.27	0.03
Device migration	1.18	0.95	1.51	0.17
Implant break	1.33	1.05	1.69	0.02
Anchor prominence	1.08	0.91	1.3	0.41
MCGR actuator malfunction	0.99	0.71	1.55	0.97
MCGR failure to lengthen	0.97	0.75	1.35	0.86
Intraoperative complications	0.95	0.74	1.31	0.74
Unexpected neurological signal chance	0.86	0.65	1.3	0.40
Neurologic injury	0.93	0.75	1.25	0.60
Pain	1.16	0.96	1.43	0.14
Pseudoarthrosis*	2.23	0.69	6.27	0.16
Wound related	1.1	0.95	1.28	0.21
Deep wound infection	1.31	1	1.76	0.06
Superficial infection	1.13	0.88	1.51	0.37
Prolonged drainage	1.22	0.8	1.98	0.42
Dehiscence	1.29	1	1.68	0.06
Unplanned surgery	1.07	0.97	1.18	0.18
Planned surgery	0.94	0.85	1.06	0.30

* Pseudoarthrosis of the proximal or distal growing rod foundations.

**TABLE 3:** The Association of BMI Status and Complications During the 2-year Follow-up

Variable	BMI status group	Total number	Rate ratio	Lower CI	Upper CI
Implant related	Underweight	9	0.45	0.20	0.90
	Healthy weight	69	0.75	0.50	1.15
	Overweight	34	Reference		
Migration	Underweight	1	0.21	0.01	1.16
	Healthy weight	18	0.84	0.38	2.04
	Overweight	8	Reference		
Implant break	Underweight	1	0.12	0.01	0.60
	Healthy weight	14	0.37	0.18	0.79
	Overweight	14	Reference		
Anchor prominence	Underweight	3	0.51	0.11	1.67
	Healthy weight	28	1.04	0.52	2.25
	Overweight	10	Reference		
MCGR actuator malfunction	Underweight	1	0.85	0.04	8.89
	Healthy weight	4	0.74	0.15	5.36
	Overweight	2	Reference		
MCGR absolute failure to lengthen	Underweight	3	1.70	0.32	9.20
	Healthy weight	6	0.74	0.20	3.52
	Overweight	3	Reference		
Intraoperative complications	Underweight	1	1.70	0.07	43.07
	Healthy weight	10	3.72	0.71	68.22
	Overweight	1	Reference		
Unexpected neurological signal change	Underweight	1	1.70	0.07	43.07
	Healthy weight	4	1.49	0.22	29.09
	Overweight	1	Reference		
Neurologic injury/impairment	Underweight	2	3.41	0.33	73.31
	Healthy weight	11	4.09	0.80	74.74
	Overweight	1	Reference		
Pain	Underweight	3	0.64	0.14	2.21
	Healthy weight	26	1.21	0.57	2.86
	Overweight	8	Reference		
Pseudoarthrosis	Underweight	0	NA		
	Healthy weight	0	NA		
	Overweight	1	NA		
Wound related	Underweight	12	0.97	0.46	1.95
	Healthy weight	30	0.53	0.31	0.94
	Overweight	21	Reference		
Deep wound infection	Underweight	2	0.43	0.06	1.70
	Healthy weight	10	0.46	0.18	1.22
	Overweight	8	Reference		
Superficial infection	Underweight	4	0.85	0.23	2.70
	Healthy weight	7	0.33	0.11	0.91
	Overweight	8	Reference		
Prolonged drainage (>7 d)	Underweight	1	0.85	0.04	8.89
	Healthy weight	4	0.74	0.15	5.36
	Overweight	2	Reference		
Dehiscence	Underweight	3	0.46	0.11	1.49
	Healthy weight	10	0.34	0.14	0.80
	Overweight	11	Reference		
Total number of complications	Underweight	51	0.65	0.47	0.89
	Healthy weight	260	0.72	0.59	0.89
	Overweight	134	Reference		
Unplanned surgical treatment	Underweight	12	0.57	0.28	1.06
	Healthy weight	89	0.92	0.63	1.37
	Overweight	36	Reference		

The EOSQ-24 domains were compared preoperatively, at 6 months, and at 2-year follow-up between the underweight, healthy, and overweight study groups (Table 4). There were no statistical differences between the study groups at any of these time points. We additionally compared the changes in the domains over time showing no differences between the weight categories.

**TABLE 4:** Patient Reported Outcome Measures using Early Onset Scoliosis Questionnaire 24 (EOSQ-24)

	Median	IQR	Significance
Preoperative			
General health			
Healthy weight (n=281)	75	25	0.926
Overweight (n=103)	75	25	
Underweight (n=58)	75	25	
Pain and discomfort			
Healthy weight (n=281)	75	38	0.684
Overweight (n=103)	75	50	
Underweight (n=58)	75	25	
Pulmonary function			
Healthy weight (n=281)	100	25	0.826
Overweight (n=103)	88	25	
Underweight (n=58)	100	34	
Transfer			
Healthy weight (n=281)	75	50	0.552
Overweight (n=103)	75	50	
Underweight (n=58)	75	50	
Physical function			
Healthy weight (n=281)	75	67	0.151
Overweight (n=103)	63	67	
Underweight (n=58)	71	67	
Daily living			
Healthy weight (n=281)	50	100	0.492
Overweight (n=103)	50	75	
Underweight (n=58)	50	63	
Fatigue and energy level			
Healthy weight (n=281)	75	50	0.454
Overweight (n=103)	75	50	
Underweight (n=58)	75	47	
Emotion			
Healthy weight (n=281)	75	38	0.159
Overweight (n=103)	75	38	
Underweight (n=58)	75	34	
Parental impact			
Healthy weight (n=281)	55	25	0.443
Overweight (n=103)	55	30	
Underweight (n=58)	60	25	
Financial impact			
Healthy weight (n=281)	75	50	0.994
Overweight (n=103)	75	50	
Underweight (n=58)	75	50	
Child satisfaction			
Healthy weight (n=281)	75	25	0.487
Overweight (n=103)	50	25	
Underweight (n=58)	75	25	
Parent satisfaction			
Healthy weight (n=281)	75	25	0.879
Overweight (n=103)	75	25	
Underweight (n=58)	75	25	
6 mo			
General health			
Healthy weight (n=221)	75	25	0.630
Overweight (n=82)	75	25	
Underweight (n=45)	75	25	
Pain and discomfort			
Healthy weight (n=221)	75	38	0.682
Overweight (n=82)	75	38	
Underweight (n=45)	75	6	
Pulmonary function			
Healthy weight (n=221)	100	25	0.858
Overweight (n=82)	100	25	
Underweight (n=45)	100	25	
Transfer			
Healthy weight (n=221)	75	50	0.568
Overweight (n=82)	75	50	
Underweight (n=45)	75	50	
Physical function			
Healthy weight (n=221)	75	58	0.334
Overweight (n=82)	67	58	
Underweight (n=45)	75	67	
Daily living			
Healthy weight (n=221)	56	84	0.181
Overweight (n=82)	50	75	
Underweight (n=45)	50	88	
Fatigue and energy level			
Healthy weight (n=221)	75	50	0.359
Overweight (n=82)	75	38	
Underweight (n=45)	75	50	
Emotion			
Healthy weight (n=221)	75	38	0.218
Overweight (n=82)	75	50	
Underweight (n=45)	88	28	
Parental impact			
Healthy weight (n=221)	60	30	0.717
Overweight (n=82)	60	30	
Underweight (n=45)	65	30	
Financial impact			
Healthy weight (n=221)	75	50	0.472
Overweight (n=82)	75	50	
Underweight (n=45)	75	25	
Child satisfaction			
Healthy weight (n=221)	75	50	0.956
Overweight (n=82)	75	25	
Underweight (n=45)	75	25	
Parent satisfaction			
Healthy weight (n=221)	75	50	0.664
Overweight (n=82)	75	50	
Underweight (n=45)	75	25	
2-y follow-up			
General health			
Healthy weight (n=206)	75	25	0.916
Overweight (n=75)	75	25	
Underweight (n=37)	75	25	
Pain and discomfort			
Healthy weight (n=206)	75	50	0.403
Overweight (n=75)	75	38	
Underweight (n=37)	75	38	
Pulmonary function			
Healthy weight (n=206)	100	25	0.346
Overweight (n=75)	88	25	
Underweight (n=37)	100	25	
Transfer			
Healthy weight (n=206)	75	50	0.625
Overweight (n=75)	75	50	
Underweight (n=37)	75	50	
Physical function			
Healthy weight (n=206)	83	58	0.252
Overweight (n=75)	67	58	
Underweight (n=37)	75	75	
Daily living			
Healthy weight (n=206)	50	78	0.769
Overweight (n=75)	50	75	
Underweight (n=37)	50	88	
Fatigue and energy level			
Healthy weight (n=206)	75	50	0.118
Overweight (n=75)	75	38	
Underweight (n=37)	75	38	
Emotion			
Healthy weight (n=206)	75	38	0.302
Overweight (n=75)	75	50	
Underweight (n=37)	88	38	
Parental impact			
Healthy weight (n=206)	60	25	0.712
Overweight (n=75)	60	30	
Underweight (n=37)	60	30	
Financial impact			
Healthy weight (n=206)	75	50	0.633
Overweight (n=75)	75	50	
Underweight (n=37)	75	50	
Child satisfaction			
Healthy weight (n=206)	75	50	0.628
Overweight (n=75)	75	25	
Underweight (n=37)	75	25	
Parent satisfaction			
Healthy weight (n=206)	75	50	0.462
Overweight (n=75)	75	38	
Underweight (n=37)	75	25	

## DISCUSSION

Overweight significantly increased the risk of rod breakage in children undergoing bilateral MCGRs implantation for early-onset scoliosis. Preoperative curve size, larger kyphosis, or etiology were not associated with this risk. In contrast to previous studies,^15^ the gain in thoracic height did not differ between the weight categories suggesting that a larger soft tissue envelope does not interfere outpatient-based distraction of the magnetically controlled growing rods.

The median gain in thoracic height over a 2-year period was relatively modest compared with previous studies utilizing traditional growing rods,^16,17^ with no significant differences observed between the weight categories. The difference between planned and achieved distraction increased over time, as shown by the linear mixed model, confirming the law of diminishing returns in this study. However, as this is a multicenter, register-based investigation, neither the planned distraction amount nor the distraction interval was standardized, which represents a limitation to this conclusion.

The statistically significant differences in BMI category distributions among the various etiological groups highlight the diverse nutritional challenges faced by these patients. Patients with neuromuscular and syndromic EOS were more likely to be underweight, reflecting the impact of their primary conditions on nutritional status and growth. In contrast, the idiopathic group had a higher proportion of healthy weight individuals, likely due to the absence of complicating comorbidities. Earlier study of 287 patients did not find an association between EOS etiology and weight percentile.^4^ Nutritional support should be tailored according to the specific needs of each patient to optimize overall health and surgical outcomes.

Patients with overweight BMI status had higher incidence of implant breakage, which may be attributed to the increased mechanical stress placed on the rods due to higher body mass and potentially greater soft tissue resistance during noninvasive lengthening procedures. This aligns with earlier studies suggesting that greater forces are required to achieve distraction in patients with increased body mass.^9,11^ Stalling and failure to lengthen typically lead to revision surgery, although overweight BMI status was not associated with unplanned revision surgeries in the present study. BMI status was not associated with differences in planned and actual distraction, with only the law of diminishing returns being observed.^18^

BMI status was not associated with EOSQ-24 scores in this study, indicating that the overall perceived well-being and function of patients and their families are not markedly affected by weight status alone. Previous studies have found etiology to be the main factor associated with patient-reported quality of life in patients with EOS.^19,20^ A previous study of 56 families and children did not find patient weight to be associated with EOSQ-24 scores.^20^

We consider the large, prospectively collected sample size of patients treated with bilateral MCGRs to be a significant strength of this study. However, it is subject to limitations inherent to all register-based studies. The study lacked detailed information on the type of skin incisions, the type of proximal anchors used, the diameter of the rods, the number of surgeons performing the index surgeries, and the specific versions of MCGRs used in this cohort. The study did not include first-generation MCGR devices. Outcomes were analyzed only for the first 2 years of treatment. This register-based study included all patients treated for early-onset scoliosis with bilateral MCGRs recorded in the study database between 2013 and 2021. During this period, multiple versions of MAGEC rods were introduced, which poses a limitation to the study. The limited statistical power prevented an analysis based on scoliosis etiology or specific individual diagnoses in the syndromic and neuromuscular groups. Although we examined the association of BMI status with outcomes in patients treated with MCGRs, other growth-friendly constructs were not evaluated. Further research is needed to explore outcomes in other constructs. In addition, disease-specific growth charts were not used for BMI calculation in syndromic or neuromuscular patients, which represents another limitation of this study. All patients did not have EOSQ-24 questionnaires at each time point limiting the comparison of health-related quality of life between the study groups.

In conclusion, although weight status does not seem to significantly impact the primary surgical outcomes of deformity correction and thoracic growth, it is associated with an increased risk of implant-related complications in overweight patients.

## COLLABORATORS

Anderson, John; Bauer, David; Cheung, Kenneth; Clement, Carter; Crawford, Haemish; Groves, Mari; Hwang, Steven; Kadado, Allen; Karlen, Judson; Kwan, Kenny; Lark, Robert; Louer, Craig; Mitchell, Stuart; Morash, Kevin; Ouellet, Jean; Segar, Anand; Shah, Suken; Shirley, Otis; Stone, Joe; Weinstein, Stuart; Wilson, Nichola; Lebel, David; Balsara, Karl; Borden, Timothy; Braga, Bruno; Buckland, Aaron; Burke, Rebecca; Drake, Luke; Fehnel, Katie; Roberto, Rolando Figueroa; Ghazi, John; Haber, Lawrence; Hughes, Michael; Iqbal, Omar; Jea, Andrew; Landrum, Matthew; Le, Hai; Lullo, Brett; Mathew, Smitha; Miller, Kyle; Montgomery, Blake; Oh, Taimin; Orland, Keith; Rushton, Paul; Shannon, Brett; Shaw, Aaron; Shea, Graham; Simmonds, Andrea; Tuason, Dominick; Van Nortwick, Sara; Bauer, Jennifer; Beauchamp, Eduardo; Bennett, James; Brockmeyer, Douglas; Bumpass, David; El-Bromboly, Yehia; Erickson, Mark; Guillaume, Tenner; Ihnow, Stephanie; Iyer, Rajiv; Jain, Viral; Kelly, Brian; Klatt, Joshua; Martin, Jonathan; McQuerry, Jessica; Miller, Daniel; Pellise, Ferran; Perra, Joseph; Price, Nigel; Schmitz, Michael; Snyder, Brian; Szczodry, Michal; Tanaka, Tomoko; Tetreault, Tyler; Smith, John; Betz, Randy; Crawford, Alvin; Demirkiran, Gokhan; Emans, John; Johnston, Charles; Karlin, Lawrence; Kawakami, Noriaki; McCarthy, Richard; Perez-Grueso, Francisco; Roye, David; Shufflebarger, Harry; PeterSturm, ; GeorgeThompson, ; Yazici, Muharrem; Accousti, William; Ahn, Edward; Akoto, Harry; Albanese, Stephen; Bellaire, Laura; Birch, Craig; Boachie-Adjei, Oheneba; Bonfield, Chris; Bouton, Daniel; Brassard, Felix; Catanzano, Anthony; Chemaly, Olivier; Cheung, Jason; Cho, Robert; Christman, Tyler; Couture, Daniel; Dahl, Benny; Devito, Dennis; Diab, Mohammad; Drake, Dan; Field, Antony; Gabos, Peter; Garcia, Alejandro Peiro; Gardner, Adrian; George, Stephen; Gerow, Frank; Gonda, David; Hammerberg, Kim; Hedequist, Daniel; Herrera, Jose; Holt, Josh; Howard, Jason; Hresko, Tim; Illingworth, Kenneth; Jones, Morgan; Kelly, Derek; Kelly, Michael; Koljonen, Paul; Labelle, Hubert; Lavelle, William; Lenke, Lawrence; Lew, Sean; Mac Thiong, Jean-Marc; Mackenzie, Stuart; MacKintosh, Erin; Mangano, Francesco; Marks, David; Marquez-Lara, Alejandro Jose; Martus, Jeffrey; Mehta, Jwalant; Metz, Lionel; Miller, Nancy; Miyanji, Firoz; Mundis, Greg; Nelson, Susan; Newton, Peter; Ede, Matthew Newton; Nguyen, Cynthia; Niazi, Toba; Pereira, Susana Nunez; Parent, Stefan; Pizones, Javier; Poon, Selina; Ramchandran, Subaraman; Ramirez, Norman; Rodriguez, Luis; Sanchez Marquez, Jose Miguel; Sanders, James; Sayama, Christina; Schulz, Jacob; Schwend, Richard; Skalak, Timothy; Speirs, Joshua; Sukkarieh, Hamdi; Thometz, John; Tice, Andrew; Upasani, Vidyadhar; Vialle, Raphael; Wang, Shengru; Warner, Bill; Wrubel, David; Wu, Nan; Yankey, Kwadwo; Zhang, Terry Jianguo; Aubin, Carl-Eric; Carry, Patrick; Mayer, Oscar; White, Rachel; Akbarnia, Behrooz; Anderson, Richard; Andras, Lindsay; Blakemore, Laurel; Cahill, Pat; El-Hawary, Ron; Flynn, Jack; Glotzbecker, Michael; Gomez, Jaime; Hardesty, Christina; Hogue, Grant; Larson, Noelle; Li, Ying; Luhmann, Scott; Murphy, Robert; Murphy, Josh; Oetgen, Matthew; Pahys, Josh; Samdani, Amer; Sawyer, Jeffrey; Skaggs, David; Sponseller, Paul; Vitale, Michael; White, Klane; Yaszay, Burt; Anari, Jason; Brooks, Jaysson; Fitzgerald, Ryan; Floccari, Lorena; Garg, Sumeet; Gupta, Purnendu; Heffernan, Michael; Helenius, Ilkka; Johnson, Megan; Matsumoto, Hiroko; McIntosh, Amy; Ramo, Brandon; Redding, Gregory; Ritzman, Todd; Rodriguez-Olaverri, Juan; Roye, Benjamin; Smit, Kevin; Swarup, Ishaan; Truong, Walter; Vorhies, John; Welborn, Michelle; Yang, Scott.
